# CSE1L is a negative regulator of the RB-DREAM pathway in p53 wild-type NSCLC and can be targeted using an HDAC1/2 inhibitor

**DOI:** 10.1038/s41598-023-43218-3

**Published:** 2023-09-27

**Authors:** Lei Duan, Mehrdad Jafari Tadi, Carl G. Maki

**Affiliations:** Department of Anatomy and Cell Biology, Rush University Medical Center, 600 S. Paulina Street, AcFac 507, Chicago, IL 60612 USA

**Keywords:** Cancer, Cell biology, Computational biology and bioinformatics, Drug discovery

## Abstract

P53 represses transcription by activating p21 expression and promoting formation of RB1-E2F1 and RBL1/RBL2-DREAM transcription repressor complexes. The DREAM complex is composed of DP1, RB-family proteins RBL1 or RBL2 (p107/p130), E2F4/5, and MuvB. We recently reported RBL2-DREAM contributes to improved therapy responses in p53 wild-type NSCLC cells and improved outcomes in NSCLC patients whose tumors express wild-type p53. In the current study we identified CSE1L as a novel inhibitor of the RBL2-DREAM pathway and target to activate RBL2-DREAM in NSCLC cells. CSE1L is an oncoprotein that maintains repression of genes that can be reactivated by HDAC inhibitors. Mocetinostat is a HDAC inhibitor in clinical trials with selectivity against HDACs 1 and 2. Knockdown of CSE1L in NSCLC cells or treatment with mocetinostat increased p21, activated RB1 and RBL2, repressed DREAM target genes, and induced toxicity in a manner that required wild-type p53. Lastly, we found high levels of CSE1L and specific DREAM-target genes are candidate markers to identify p53 wild-type NSCLCs most responsive to mocetinostat. Thus, we identified CSE1L as a critical negative regulator of the RB-DREAM pathway in p53 wild-type NSCLC that can be indirectly targeted with HDAC1/2 inhibitors (mocetinostat) in current clinical trials. High expression of CSE1L and DREAM target genes could serve as a biomarker to identify p53 wild-type NSCLCs most responsive to this HDAC1/2 inhibitor.

## Introduction

Wild-type p53 is a stress-responsive transcription factor and potent tumor suppressor^1–8^. p53 activates or represses genes involved in cell cycle progression or apoptosis to arrest proliferation or induce cell death. Transcription repression by p53 is indirect. Thus, p53 induces expression of p21 that inhibits cyclin-CDK complexes, leading to hypophosphorylation of RB family proteins RB1, RBL1, and RBL2. Hypophosphorylated RB proteins then bind E2F factors to form RB1-E2F1 and RBL1/2-DREAM transcription repressor complexes (DREAM stands for (**D**P, **R**B-related, **E**2F **a**nd **M**uvB)^9^. We recently reported RBL2-DREAM-mediated repression of aurora kinase (AURK) pathway genes improves therapy responses in p53 wild-type (WT) non-small cell lung cancer (NSCLC) cells, and loss of this repression correlates with poor outcomes specifically in patients whose tumors express WT p53^10^. Thus, transcription repression by RB-DREAM is important for p53-mediated tumor suppression in NSCLC. Therapies that activate the RB-DREAM pathway are predicted to improve clinical outcomes in p53 WT NSCLC patients.

The cell-cycle regulators CDK4 and CDK6 mediate phosphorylation of RB family proteins. This phosphorylation releases inhibition of E2F transcription factors, which then activate genes whose protein products promote DNA replication and cell progression into S phase. Inhibition of CDK4/6 leads to hypophosphorylated RB that inhibits E2F-mediated transcription, leading to cell cycle arrest^11^. CDK4/6 inhibitors palbociclib, ribociclib, and abemaciclib are approved for treatment of postmenopausal women with advanced ER + /HER2− breast cancer^11,12^. However, CDK4/6 inhibitors failed to show significant effect in NSCLC in a number of randomized clinical trials^13–15^, likely due to inefficient RB activation in some tumors and lack of markers to preselect responsive patients. Thus, there is a need to develop new RB-DREAM targeted therapy strategies and predictive biomarkers for response.

In the current study we identified CSE1L (chromosome segregation 1 like) as a novel inhibitor of RB-DREAM and potential target for its reactivation in p53 WT NSCLCs. CSE1L was reported to maintain epigenetic gene silencing, and depletion of CSE1L reactivated methylated genes that were also reactivated by treatment with histone deacetylase inhibitors (HDACi)^16^. The HDAC1/2 inhibitor mocetinostat is in current clinical trials for NSCLC and other cancers. We found that CSE1L knockdown or mocetinostat treatment increased p21, activated RB-DREAM, and induced toxicity in p53 WT but not p53 null NSCLC cells. Importantly, the p21 increase in CSE1L knockdown and mocetinostat treated cells occurs in a p53-dependent way, but independent of p53 levels. Lastly, we compared mocetinostat sensitivity in a panel of p53 WT NSCLC cell lines and cross-referenced the findings with gene expression profiles in the Cancer Cell Line Encyclopedia. Cell lines with high expression of CSE1L and AURK pathway genes are more sensitive to mocetinostat than cell lines with low expression of these genes. These findings suggest high expression of CSE1L and specific RB-DREAM target genes could serve as a clinical marker to identify p53 WT NSCLC tumors most responsive to mocetinostat.

## Results

**Figure 1:**
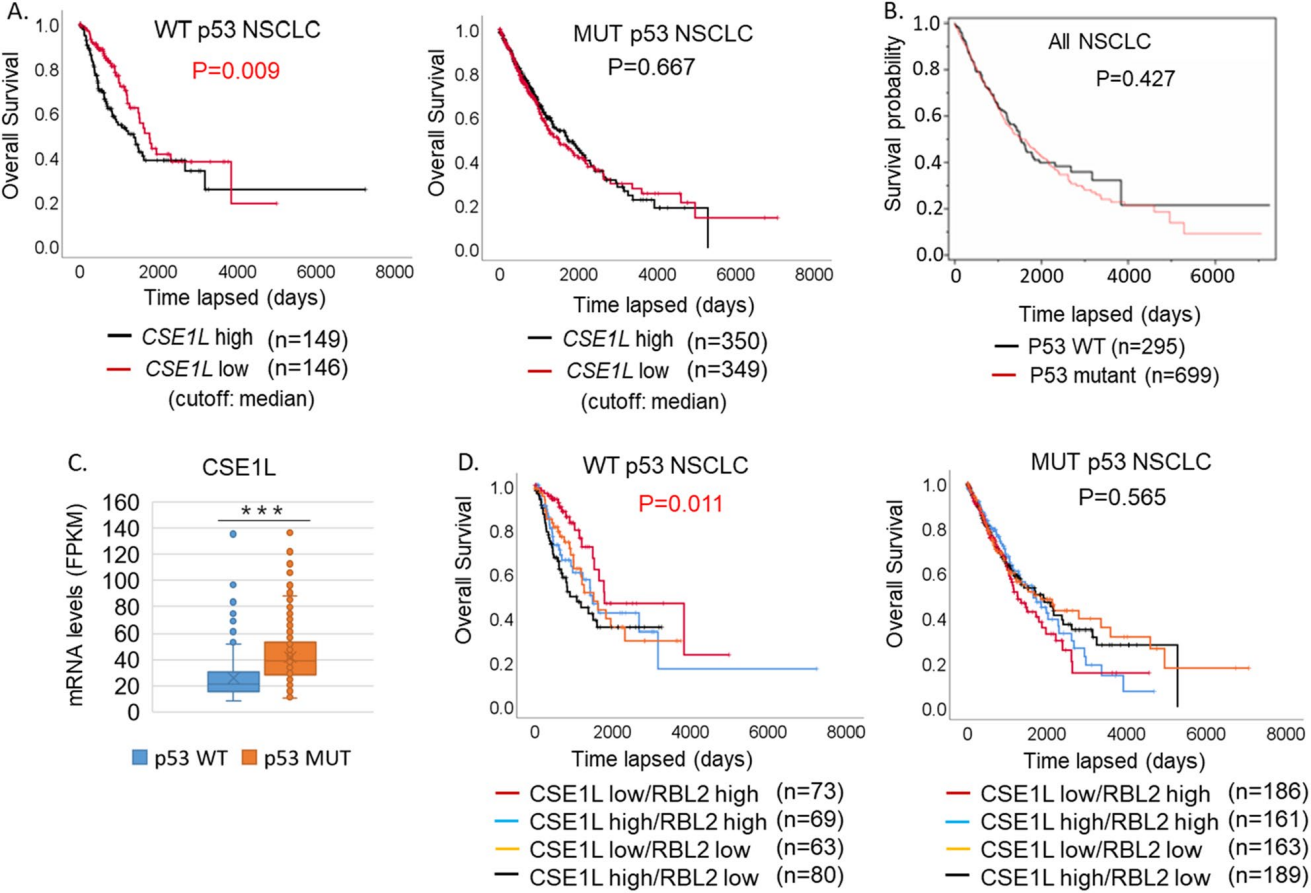
High expression of CSE1L correlates with worse prognosis in p53 WT NSCLC. Expression of CSE1L gene is tested for correlation with survival in all 994 NSCLC patients in TCGA. (**A**) High expression (≥ median) of *CSE1L* significantly (*p* = 0.009) associates with worse survival in WT p53 patients but not (*p* = 0.667 mutant p53 mutant patients. (**B**). p53 WT and p53 mutant NSCLC patients were compared for overall survival. There is no significant difference between p53 WT and p53 mutant cases. (**C**). CSE1L mRNA levels (FPKM) from TCGA in p53 WT tumors and p53 mutant tumors were compared. There is significant difference (*p* = 0.0001) between p53 WT and p53 mutant cases. (**D**). Patients were further stratified into four groups: CSE1L high/RBL2 low, CSE1L high/RBL2 high, CSE1L low/RBL2 low, and CSE1L low/RBL2 high. There is a significant difference between the groups in WT p53 patients (p = 0.011) but not mutant p53 patients (*p* = 0.565).

In search for factors that regulate the RB-DREAM pathway, we analyzed TCGA database for genes that negatively correlate with *RBL2* and *CDKN1A* (p21) and positively correlate with DREAM-target genes in NSCLC tumors. We identified *CSE1L* that positively correlates with DREAM-target genes and negatively correlates with *RBL2* and *CDKN1A* but not with *RB1* and *RBL1* (Table 1). Notably, the correlation coefficients (R) are higher in p53 WT NSCLC than in p53 mutant NSCLC. Previously we reported that high levels of RB-DREAM target gene expression correlates with poorer outcomes in p53 WT NSCLC but not p53 mutant NSCLC^10^. Thus, we speculated high levels of *CSE1L* may also correlate with poor outcomes specifically in p53 WT cases. No difference in outcomes was observed when comparing p53 WT and p53 mutant NSCLC cases directly (Fig. 1B). However, consistent with our hypothesis, we found high levels of *CSE1L* significantly correlate with poor outcomes in p53 WT NSCLC patients but not p53 mutant (Fig. 1A). Interestingly, CSE1L expression is higher in p53 mutant tumors than that in p53 WT tumors (Fig. 1C), suggesting CSE1L may be repressed by p53. We also reported that high levels of RBL2 correlate with better outcomes in p53 wild-type NSCLC. In this current analysis, we further stratified NSCLC patients into four subgroups based on their combined levels of CSE1L and RBL2. The results revealed that patients with high levels of CSE1L and low levels of RBL2 had the worst prognosis, while those with low levels of CSE1L and high levels of RBL2 had the best prognosis (Fig. 1D). Again, this was true in p53 WT but not p53 mutant cases (Fig. 1D).

**Table 1:** Pearson coefficient between CSE1L and genes of interest.

	Pearson coefficient (R)	
	p53 WT	p53 MUT
	CSE1L	CSE1L
AURKA	0.63	0.56
AURKB	0.58	0.52
BUB1	0.69	0.56
BUB1B	0.74	0.54
CCNA2	0.64	0.52
CCNB1	0.59	0.52
CCNB2	0.70	0.49
CDK1	0.69	0.46
PLK1	0.66	0.55
PLK4	0.66	0.45
RB1	0.38	0.15
RBL1	0.72	0.52
RBL2	− 0.16	− 0.15
CDKN1A	− 0.17	− 0.12

CSE1L positively correlates with DREAM-target genes and negatively with RBL2 and CDKN1A in NSCLC (TCGA). Gene expression (FPKM) in p53 WT (n = 295) and p53 MUT (n = 699) NSCLC was extracted from the TCGA database. Correlation between CSE1L and the indicated genes was analyzed with Pearson with Pearson coefficient indicated.

**Figure 2:**
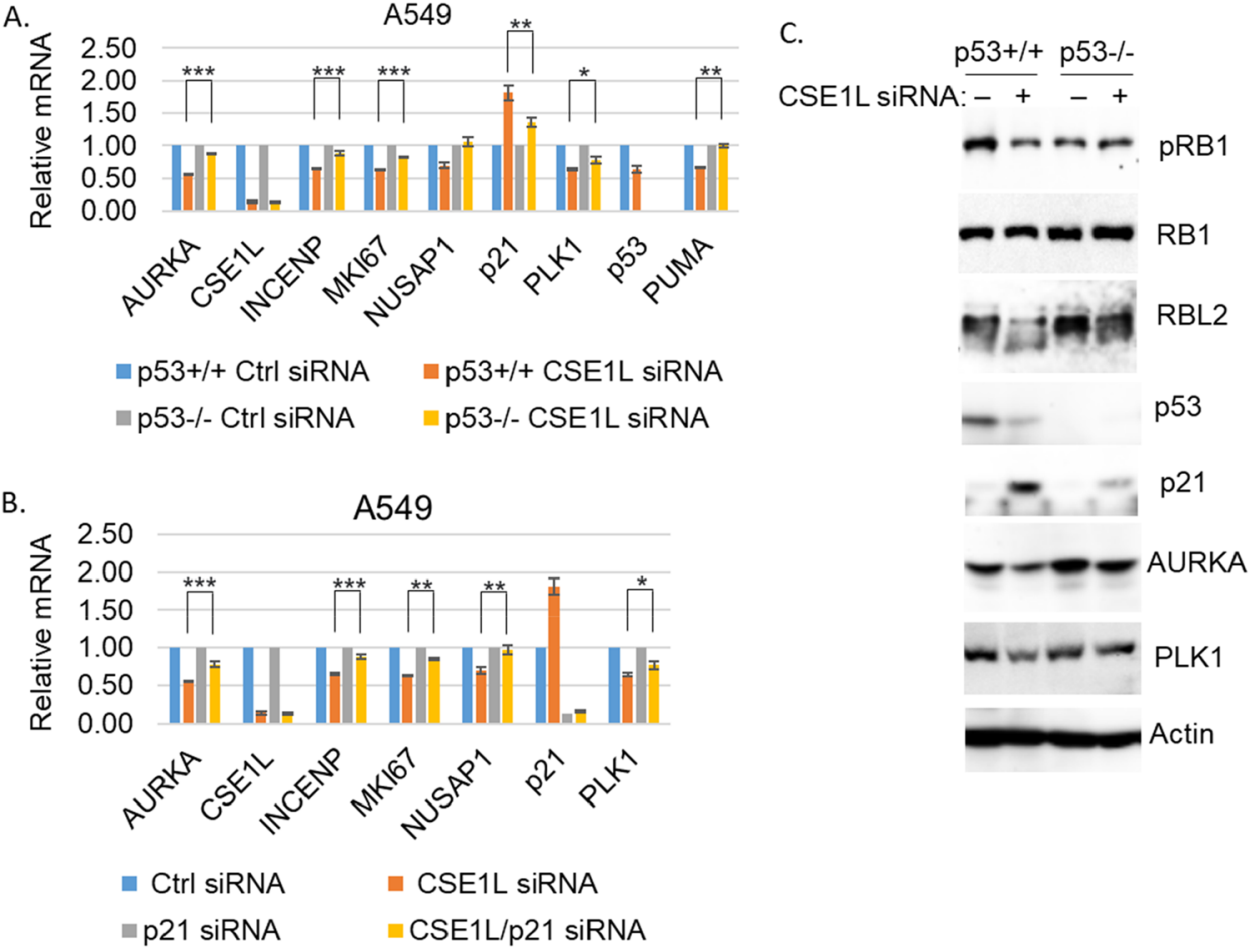
Depletion of CSE1L induces a p53/p21 dependent downregulation of AURKA pathway genes and activation of RB. A549 p53+/+ and p53−/− cells were transfected with control siRNA or CSE1L RNA for 48 h. mRNA was qPCR analyzed for the indicated genes. Average (from three experiments) relative mRNA is presented with SD indicated. Statistical differences are indicated (* *p* < 0.05, ** *p* < 0.01, *** *p* < 0.001) (**A**). Lysates were immunoblotted for the indicated proteins (representative images of three independent experiments) (**C**). (**B**) A549 p53+/+ cells were transfected with control siRNA, CSE1L RNA, and/or p21 siRNA for 48 h. mRNA was qPCR analyzed for the indicated genes. Average (from three experiments) relative mRNA is presented with SD indicated. Statistical differences are indicated.

CSE1L is reported to maintain epigenetic gene silencing and depletion of CSE1L reactivated methylated genes that were also reactivated by treatment with histone deacetylase inhibitors (HDACi)^16^. Since CSE1L positively correlates DREAM-target genes, we hypothesized CSE1L may regulate their expression. To test this, we transfected p53+/+ A549 and p53−/− A549 cells^10^ with control siRNA or CSE1L siRNA and assessed expression of a number of DREAM- targets. The results showed CSE1L knockdown decreased the tested DREAM-target genes (*AURKA, INCENP, MKI67, NUSAP1, PLK*) and this effect was significant in p53+/+ A549 cells but not in p53−/− cells, indicating p53 plays a role (Fig. 2A). We speculated CSE1L knockdown may reduce DREAM-target genes by increasing p21, as CSE1L negatively correlates with p21 (CDKN1A) in NSCLC (Table 1). Indeed, p21 mRNA and protein were increased by CSE1L knockdown that was more pronounced in p53+/+ A549 cells (Fig. 2A and C). CSE1 L knockdown also caused hypophosphorylation of RB1 and RBL2, evidenced by decreased levels of phospho-RB1 and appearance of faster migrating RBL2 protein, and this was largely p53 dependent (Fig. 2C). Interestingly, p53 mRNA and protein were not increased but rather decreased by CSE1L knockdown even though p21 was increased in a p53-dependent way (Fig. 2A, C). This indicates the increase in p21 is not because p53 protein levels increased. Notably, the direct p53 target PUMA was not increased but rather decreased in p53+/+ cells upon CSE1L knockdown, consistent with the decrease in p53 levels. Thus, the increased expression in CSE1L knockdown cells is specific to p21 and not all p53 target genes. To test if p21 is required for the CSE1L knockdown effect, we co-transfected A549 p53+/+ cells with CSE1L and p21 siRNA. Knockdown of p21 significantly restored expression of the DREAM-target genes in CSE1L knockdown cells (Fig. 2B), indicating repression of the genes is p21-dependent. Thus, the results support a model in which CSE1L promotes expression of DREAM-target genes by repressing the p21-RB-DREAM pathway. Dong et al. reported that the knockdown of CSE1L led to the reactivation of methylated genes, a response also observed with HDAC inhibitors^16^. Additionally, CSE1L was found to promote the nuclear import of HDAC1, 2, and 8. To investigate whether CSE1L regulates the nuclear import of HDACs in A549 cells, we transfected the cells with either control siRNA or CSE1L siRNA. Subsequently, cytoplasmic/nuclear fractionation was performed, followed by immunoblotting for HDAC1. The results revealed consistent activation of RB1 upon CSE1L knockdown. However, the distribution of HDAC1 proteins between the cytoplasm and nucleus showed similar patterns in both the control siRNA and CSE1L siRNA cells (Fig. S2). This finding suggests that CSE1L does not suppress RB by promoting the nuclear import of HDAC1 in these cells.

**Figure 3:**
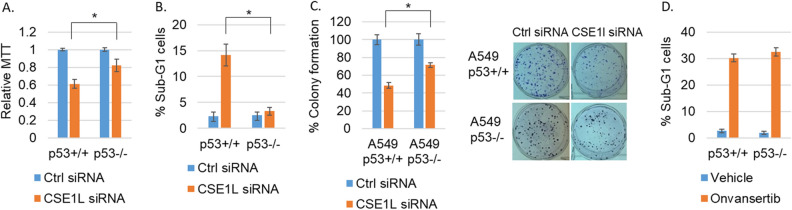
Depletion of CSE1L induces a more pronounced cytotoxicity in p53 WT NSCLC cells. A549 p53+/+ and p53−/− cells were transfected with control siRNA or CSE1L siRNA for 72 h and then analyzed with MTT assay (**A**) or FACS analysis for sub-G1 (**B**). Average (8 replicates) relative MTT absorbance and average (triplicate) % sub-G1 cells are presented with SD indicated. (**C**) A549 p53+/+ and p53−/− cells were transfected with control siRNA or CSE1L siRNA for 48 h and then plated for colony formation assay. Average (triplicate) % colony formation is presented with SD indicated. Representative images are presented. (**D**). A549 p53+/+ and p53−/− cells were treated with vehicle or onvansertib (1 µM) for 72 h and then analyzed for sub-G1 cells. % sub-G1 cells are presented with SD indicated. Statistical differences (* *p* < 0.05) are indicated in (**A**), (**B**), and (**C**). There is no significant difference in D.

Finally, we tested the effect of CSE1L knockdown on cell viability using MTT assay, FACS analysis of sub-G1 cells for apoptosis, and long-term colony formation. The results showed CSE1L knockdown decreased viability and survival and increased apoptosis in a manner that was largely p53 dependent (Fig. 3A–C). Notably, the PLK1 specific inhibitor onvansertib decreased viability and induced apoptosis similarly in p53+/+ and p53−/− A549 cells (Fig. 3D), suggesting the apoptotic effect of CSE1L knockdown in p53+/+ A549 cells but not in p53−/− cells could be through downregulating DREAM-target genes such as PLK1 rather than p53-mediated activation of apoptotic genes (PUMA is not induced). Altogether, these results establish CSE1L as an inhibitor of p21-DREAM pathway that promotes expression of DREAM-target genes and cancer progression, especially in p53 WT NSCLC.

**Figure 4:**
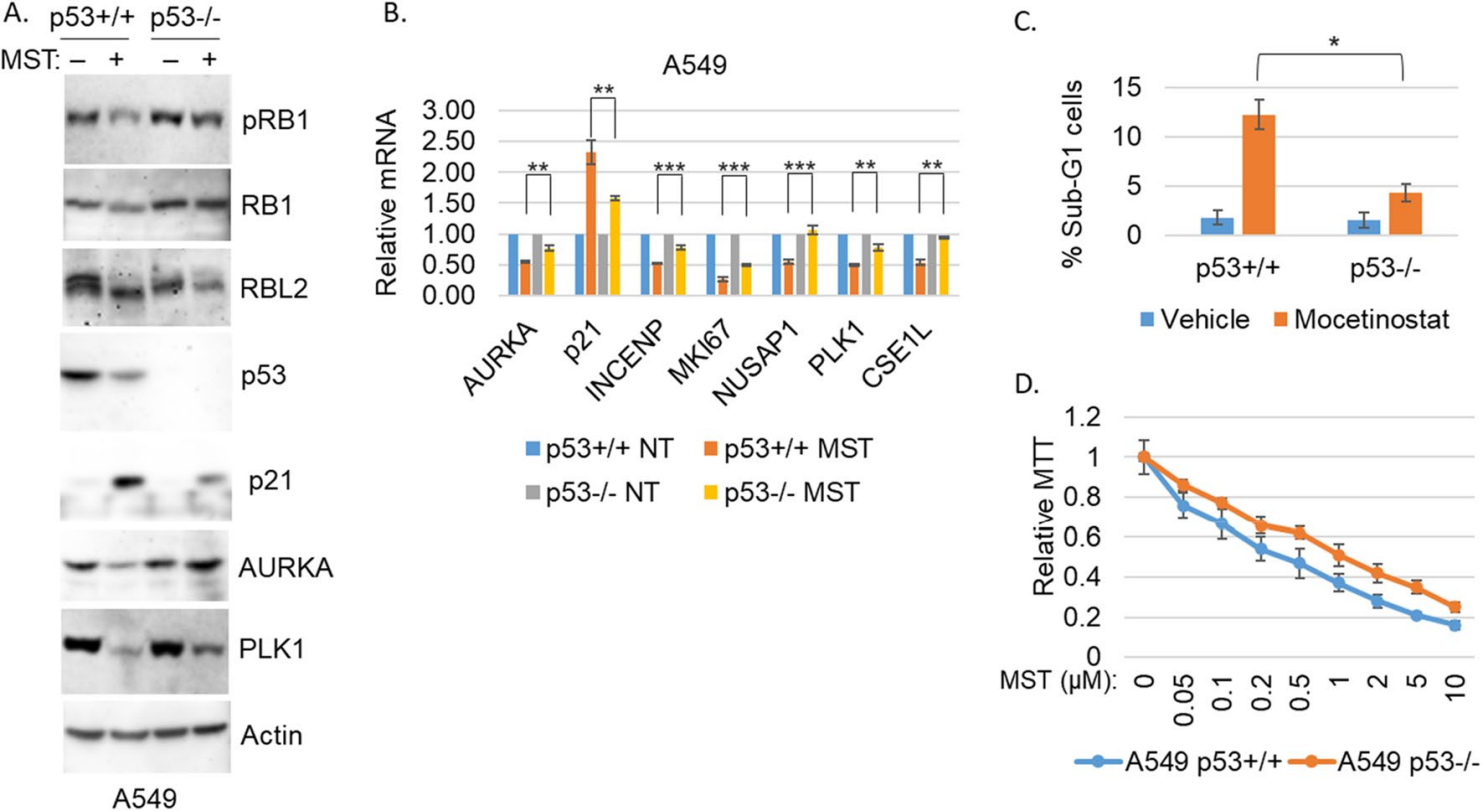
p53 WT cells are more sensitive to mocetinostat. A549 p53+/+ and p53−/− cells were treated with vehicle or mocetinostat (1 µM) for 24 h. Lysates were immunoblotted for the indicated proteins (representative images of three independent experiments) (**A**). mRNA was qPCR analyzed for the indicated gene. Average (from three experiments) relative mRNA is presented with SD indicated. Statistical differences are indicated (*p < 0.05, **p < 0.01, ***p < 0.001) (**B**). (**C**) A549 p53+/+ and p53−/− cells were treated with vehicle or mocetinostat (1 µM) for 72 h and then analyzed for sub-G1 cells. % sub-G1 cells are presented with SD indicated. Statistical difference is indicated (*p < 0.05). (**D**). A549 p53+/+ and p53−/− cells were treated with vehicle or the indicated doses of mocetinostat (1 µM) for 72 h and then analyzed with MTT assay. Average (8 replicates) relative MTT absorbance is presented with SD indicated.

CSE1L knockdown and HDAC inhibitors activated similar sets of genes, and HDACs are known to repress p21^16,17^. Thus, we hypothesized inhibition of HDACs may be a way to activate the RB-DREAM that is inhibited by CSE1L. Mocetinostat is a HDAC1/2 inhibitor in current clinical trials (NCT02954991, NCT02805660) for NSCLC. To test our hypothesis, we treated p53+/+ A549 and p53−/− A549 cells with mocetinostat and monitored gene and protein expression as well as cell survival. Similar to CSE1L knockdown, mocetinostat increased p21 expression and induced hypophosphorylation of RB1/RBL2 (Fig. 4A), and repressed DREAM-target genes and proteins (Fig. 4A and B). Importantly, these effects of mocetinostat were more pronounced in p53+/+ A549 cells than p53−/− A549 cells. P53 protein was decreased by mocetinostat (Fig. 4A) even though p21 was increased in a largely p53-dependent way, similar to CSE1L knockdown. Lastly, mocetinostat also induced loss of viability and apoptosis that was more pronounced in p53+/+ A549 cells than p53−/− A549 cells (Fig. 4C and D). These results suggest HDAC1/2 inhibitors may be used to activate the CSE1L-repressed RB-DREAM pathway in p53 WT NSCLC cells.

**Figure 5:**
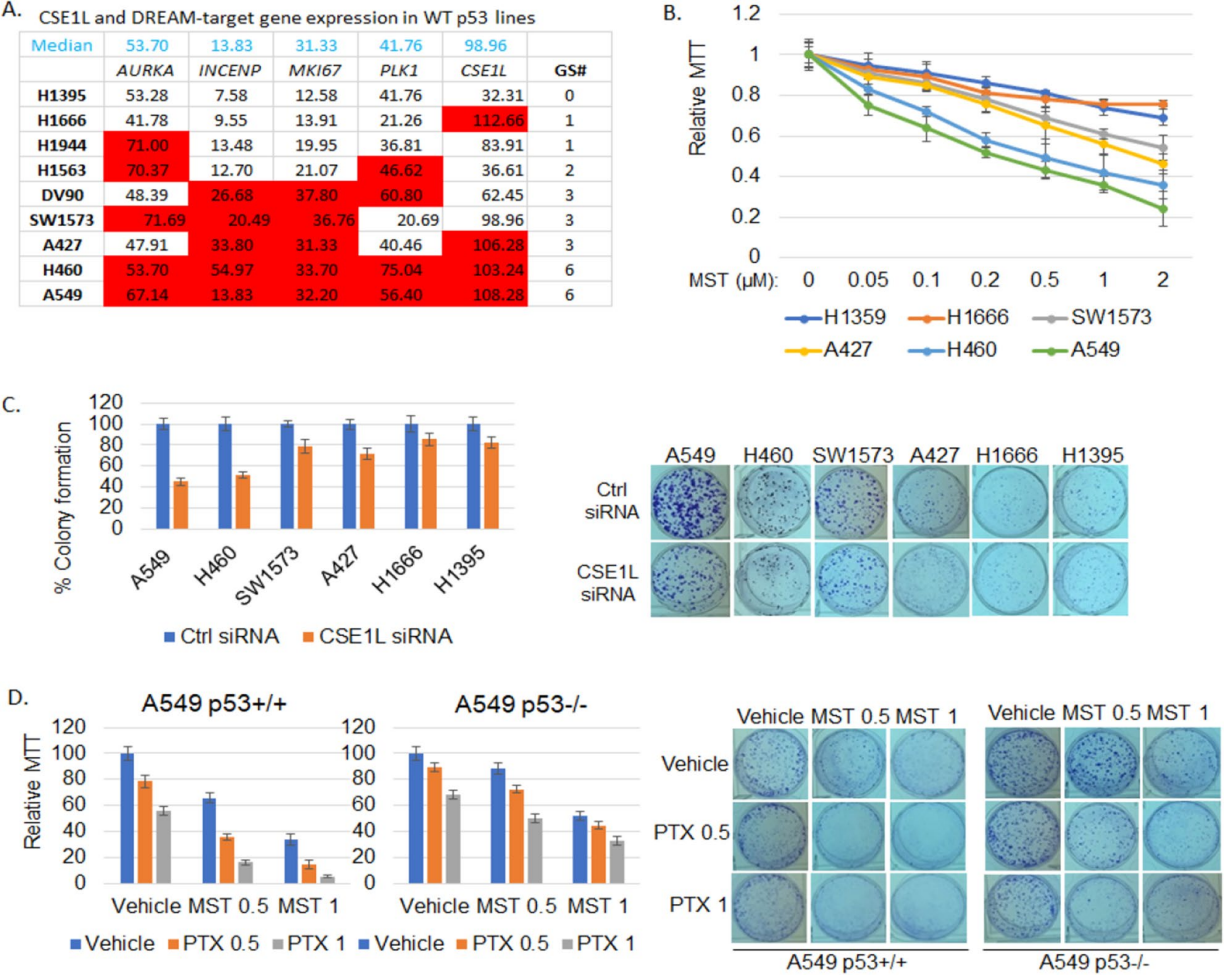
CSE1L and AURKA pathway gene high p53 WT NSCLC cell lines are more sensitive to mocetinostat and CSE1L depletion. (**A**) The gene expression of CSE1L and the AURKA pathway in 9 p53 WT NSCLC cell lines was extracted from the CCLE RNAseq database. The median expression level of each gene was utilized as a cutoff (1 or 0) to determine high or low expression. The cell lines were then ranked based on the sum of the genes' high expression levels, referred to as the gene set score (GS#). (**B**) The indicated cell lines were treated with vehicle or the indicated doses of mocetinostat (1 µM) for 72 h and then analyzed with MTT assay. Average (8 replicates) relative MTT absorbance is presented with SD indicated. (**C**) The indicated cell lines were transfected with control siRNA or CSE1L siRNA for 48 h and then plated for colony formation assay. Average (triplicate) % colony formation is presented with SD indicated. Representative images are presented. There are significant differences (*p* < 0.05) between A549/H460 and the other four cell lines in CSE1L treated conditions. (**D**) A549 p53+/+ and p53−/− cells were treated with vehicle or the indicated doses of mocetinostat (0.5 µM or 1 µM) and/or PTX (0.5 nM or 1 nM) for 72 h and then grown in drug free media for colony formation assay. Average (triplicate) % colony formation is presented with SD indicated. Representative images are presented. There is a synergistic effect of mocetinostat and PTX in p53+/+ cells (CI < 1) but not in p53−/− cells (CI > 1) (see CompuSyn analysis in Fig. S7).

We recently reported p53 WT NSCLC cells expressing high levels of aurora kinase pathway genes (DREAM-target genes) are more susceptible to aurora kinase inhibitors^10^. We speculated p53 WT NSCLC cells expressing high levels of CSE1L and DREAM-target genes would be more sensitive to mocetinostat. To test this, we first compared mRNA levels of CSE1L, AURKA, INCENP, MKI67, NUSAP1, and PLK1 in 9 p53 WT NSCLC cell lines using the Cancer Cell Line Encyclopedia (CCLE) RNAseq database (Fig. 5A). A549 and H460 cells express higher levels of CSE1L and DREAM-target genes indicated with higher GS# score (number of genes whose levels > median) while H1395 and H1666 cells express lower CSE1L and DREAM-target genes (GS# scores are lower). We treated these cells with increasing doses of mocetinostat and analyzed viability by MTT assay. A549 and H460 cells are significantly more sensitive to mocetinostat than H1395 and H1666 cells and two other cell lines (A427, SW1573) that have intermediate GS# scores (Fig. 5B). Furthermore, A549 and H460 cells were also more dependent on CSE1L as depletion of CSE1L caused a greater reduction in colony formation in these cells compared to the other four cell lines (Fig. 5C). This suggests WT p53 NSCLC cells that express high levels of CSE1L and DREAM target genes may be more susceptible to targeting CSE1L-mediated RB-DREAM pathway with HDAC1/2 specific inhibitors.

Previously we showed that inhibition of one RB-DREAM target (AURKA) sensitizes NSCLC cells to paclitaxel in a p53-dependent manner. Because mocetinostat downregulates AURKA and AURKA.

pathway genes, we asked if mocetinostat can sensitize NSCLC cells to paclitaxel and if the effect is p53 dependent. To test this, A549 p53+/+ and A549 p53−/− cells were treated with different doses of mocetinostat and/or paclitaxel and cell survival was evaluated by colony formation. The results showed that there is a synergistic effect of mocetinostat combination with paclitaxel in A549 p53+/+ cells but not in A549 p53−/− cells (Figs. 5D and S7).

## Discussion

Our recent studies showed the RBL2-DREAM pathway contributes to p53-mediated tumor suppression in NSCLC^10^. Thus, high expression of RBL2 and reduced expression of DREAM-target genes (AURK pathway genes) associated with improved outcomes in p53 WT but not p53 mutant NSCLC patients. Moreover, NSCLC cell lines with high expression of RBL2 and reduced expression of AURK pathway genes showed improved responses to chemotherapy and radiation in the p53 WT setting. Accordingly, treatments that can activate RB-DREAM are predicted to improve therapy responses and outcomes in p53 WT NSCLC patients and are therefore worth pursuing. In the current study we identified CSE1L as a novel inhibitor of RB-DREAM and target for its activation in p53 WT NSCLCs. Direct inhibition of CSE1L by gene knockdown or inhibition of HDAC1/2 by mocetinostat treatment activated RB-DREAM and induced toxicity in p53 WT but not p53 mutant NSCLC cells.

CSE1L was recently identified in a screen for factors that maintain repression of DNA methylated genes^16^. In that study, knockdown of CSE1L increased expression of multiple genes that were otherwise silenced by DNA methylation. Interestingly, increased expression of these genes upon CSE1L knockdown did not result from reduced DNA methylation, but instead appeared to result from decreased nuclear import of histone deacetylases HDACs 1, 2, and 8. Thus, most of the DNA methylated genes that were reactivated upon CSE1L knockdown were also reactivated by HDAC inhibitors TSA and SAHA. These results supported a model in which CSE1L represses expression of methylated genes potentially by promoting nuclear import of HDACs 1, 2, and 8. In the current report, we found CSE1L knockdown or treatment with the HDAC1/2 inhibitor mocetinostat similarly increased p21 mRNA and protein expression to activate the RB-DREAM pathway, indicating that CSE1L-mediated repression of the p21-RB pathway can be overcome by HDAC1/2 inhibitors. However, our experiments did not corroborate the findings of Dong and Zhu et al. regarding the role of CSE1L in promoting the nuclear import of HDAC1 in A549 cells. This suggests the possibility of alternative mechanisms through which CSE1L regulates the silencing of the p21 gene. It is unclear whether the p21 gene is methylated in our studies, though inhibition of the DNA methyltransferase DNMT1 was previously reported to increase p21 expression^18^. Importantly, we found the p21 increase in CSE1L knockdown and mocetinostat treated cells occurs in a p53-dependent way, but independent of p53 levels (p53 levels did not increase). Previous studies reported p53 binds the p21 gene promoter in unstressed cells, and then activates p21 gene expression in response to chemotherapy stress or other stimuli^19^. Therefore, p53 may be bound to the p21 promoter in our studies and only activate p21 expression when HDACs are inhibited. An alternative is that CSE1L knockdown or HDAC inhibition increases p53 binding to the p21 gene promoter without increasing p53 protein levels.

Previous studies reported a correlation between high expression of CSE1L and poor prognosis in non-small cell lung cancer (NSCLC)^20^. Our analysis of the KM plotter Gene-Chip database confirmed that high CSE1L expression is associated with significantly decreased overall survival (OS) in 2166 cases of NSCLC (Fig. S1), without taking into consideration the p53 status. However, analysis of the TCGA database revealed that CSE1L correlates with poorer prognosis only in p53 wild-type NSCLC but not in p53 mutant NSCLC (Fig. 1). Furthermore, our analysis found that when combined with RBL2 levels, patients with high levels of CSE1L and low levels of RBL2 had the worst outcomes, while those with low levels of CSE1L and high levels of RBL2 had the best prognosis. This association was observed in a p53-dependent manner, specifically in wild-type p53 cases. The results suggest that the CSE1L-regulated RB-DREAM pathway is particularly critical for p53 wild-type tumor response to therapy and prognosis. This is consistent with our findings that the RB-DREAM pathway represses AURKA pathway gene expression, which is critical for therapy response in a p53-dependent pathway^10^.

Mocetinostat is in current clinical trials for NSCLC. In our recent study we found NSCLC cell lines with low RBL2 expression and high expression of AURK pathway genes had heightened sensitivity to AURK pathway inhibitors alone or with paclitaxel (PTX), a standard NSCLC chemotherapy agent. Results from the current study show CSE1L represses RB/RBL2 activity to maintain AURK pathway genes, and that CSE1L knockdown or mocetinostat treatment reverses these effects. Therefore, we hypothesized high expression of CSE1L and AURK pathway genes may identify p53 WT NSCLC cells most susceptible to mocetinostat or the clinically relevant mocetinostat plus PTX combination. We tested this hypothesis by generating a gene signature score in p53 WT NSCLC cell lines based on whether CSE1L and AURK pathway gene expressions were above or below the median. Consistent with our hypothesis, the results showed cell lines with high CSE1L and AURK pathway gene expression (high gene signature scores) were more sensitive to mocetinostat than cell lines with low gene signature scores. The results imply that a gene signature based on CSE1L and AURK pathway gene expression similar to the one we developed here could be used to identify p53 WT NSCLC tumors that will be most sensitive/responsive to mocetinostat plus PTX. This has clear clinical implications since there are no molecular markers currently to identify NSCLCs that will or will not respond to this drug combination. It is worth noting that CSE1L itself is a gene targeted by RB-DREAM^9^ and is downregulated by mocetinostat (Fig. 4B). CSE1L regulates the nuclear transport of p65 that are important for NSCLC^20^. Thus, the effect of mocetinostat is likely twofold; it inhibits HDACs to downregulate CSE1L to suppress other nuclear factors.

In summary, our study has identified CSE1L as a critical inhibitor of the RB-DREAM repressor complex and highlights HDAC1/2 as a target in the CSE1L-RB-DREAM pathway. Our findings also suggest that p53 wild-type NSCLC cells that exhibit elevated expression levels of CSE1L and AURKA pathway genes may be particularly responsive to the HDAC1/2 inhibitor mocetinostat.

## Methods

### Cell lines and reagents

NSCLC cell lines H1666, A427, A549, H1395, SW1573 and H460 are acquired from ATCC. Crispr p53 KO A549 (p53−/−) cells and control parental A549 (p53 +/+) cells are a generous gift from Dr. William Hahn^21^ (Dana-Farber Cancer Institute). Cells were grown in RPMI medium, with 10% fetal bovine serum (FBS), penicillin (100 U/mL) and streptomycin (100 µg/mL). Mocetinostat, onvansertib and paclitaxel were obtained from Selleck Chemicals.

### TCGA and CCLE RNAseq databases and bioinformatics analysis

Analysis of TCGA and CCLE RNAseq databases are previously described^10^. Briefly, P53 mutation status was obtained from the Pan-lung TCGA^22^ in cbioportal (www.Cbioportal.org). NSCLC Gene expression and survival data were downloaded from proteinatlas (proteinatlas.org/pathology/lung + cancer) as we previously described^10^. All 994 patients from this dataset were tested for association of p53 status or gene expression levels with survival by Kaplan–Meier curve (IBM SPSS). Patients with ≥ median gene expression levels (FKPM) are taken as high expression (~ 50% of patients) and < median are taken as low expression. Gene expression correlation is analyzed with Pearson co-efficiency using Microsoft Excel.

CCLE RNAseq gene expression (RPKM) database^23^ for 1019 cell lines (CCLE RNAseq genes rpkm 20180929.gct.gz) was downloaded from https://portals.broadinstitute.org/ccle/data under Current Data. The CSE1L and RBL2 gene expression was extracted from the database (Tables S1 and S2).

### Immunoblotting

Immunoblotting was done as described previously^10^. Whole cell extracts were prepared by scraping cells in lysis buffer (150 mM NaCl, 5 mM EDTA, 0.5% NP40, 50 mM Tris, pH 7.5), resolved by sodium dodecyl sulfate polyacrylamide gel electrophoresis (SDS-PAGE) and transferred to polyvinylidene difluoride membranes (Thermo Fisher Scientific). Antibodies to p53 (DO-1, SC-126) and β-actin (C4, SC-47778) were from Santa Cruz. Antibodies phospho-RB1 (S807/811, #8516), RB1 (#9309), RBL2 (#13610) AURKA (#14475), PLK1 (#4535), and p21 (#2947) were from Cell Signaling. Primary antibodies were detected with goat anti-mouse or goat anti-rabbit secondary antibodies conjugated to horseradish peroxidase (Life Technologies), using Clarity chemiluminescence (BIO-RAD). Digital images were acquired using a UVP multispectral bioimaging system and saved as TIFF files. The presented blot bands were cropped from the original images using Photoshop software.

### siRNA-mediated transient Knockdown

Pooled CSE1L siRNA, p21 siRNA (On-target plus smart pool) and Control siRNA (On-target plus siControl non-targeting pool) were purchased from Dharmacon. Cells were transfected according to the manufacturer's guidelines using DharmaFECT I reagent.

### Subcellular protein fractionation

Cells were fractionated for subcellular proteins using Subcellular Protein Fractionation Kit from ThermoFisher Scientific (Waltham, MA) as previously described^24^. Cytoplasmic and nuclear proteins were isolated following the protocol from the manufacturer.

### RNA isolation and Real-time quantitative PCR analysis

RNA isolation and qPCR analysis was done as described previously^10^. Total RNA was prepared using Total RNA Mini Kit (IBI Scientific, IA); the first cDNA strand was synthesized using High Capacity cDNA Reverse Transcription Kit (Applied Biosystems, CA). Manufacturers’ protocols were followed in each case. The PCR primers for the indicated genes are listed in Table S3. SYBR green PCR kit (Applied Biosystems) was used according to the manufacturer’s instructions. AB7300 system was used as follows: activation at 95 °C; 2 min, 40 cycles of denaturation at 95 °C; 15 s and annealing/extension at 60 °C; 60 s, followed by melt analysis ramping from 60 to 95 °C. Relative gene expression was determined by the ΔΔC_t_ method using β-Actin to normalize. PCR reaction was conducted in technical triplicate and average CT values were used to calculate relative expression of genes.

### Colony formation assay

Cells were plated in 6-well plates with 200 cells/well in triplicate for 24 h. Cells were then transfected with control siRNA or CSE1L siRNA for 24 h or treated with the indicated drugs for three days and then released of drugs. Cell were allowed to recover for 2–5 weeks to form colonies. Colonies were stained with 1% methylene blue (Sigma) in ethanol and number of positive colonies was counted. Experiments are conducted in triplicate and repeated at least one more time. Average value from one representative experiment is presented with SD indicated as error bars. For siRNA transfected cells, cells were detached 24 h after transfection and then plated accordingly for different treatments.

### Statistical analysis

One-way analysis of variance (ANOVA) and Student's *t*-test were used to determine the statistical significance of differences among experimental groups. Student's *t*-test was used to determine the statistical significance between control and experimental groups. For Kaplan–Meier survival analysis, Log-Rank test was used to determine significance between patient groups with high or low expression of the genes.

### Supplementary Information

Supplementary Figures.

Supplementary Tables.

## Data Availability

All data generated or analyzed during this study are included in this published article [and its supplementary information files].
