# Supplementary material for: CSE1L is a negative regulator of the RB-DREAM pathway in p53 wild-type NSCLC and can be targeted using an HDAC1/2 inhibitor
Source: Sci Rep. 2023 Sep 27;13:16271. doi: 10.1038/s41598-023-43218-3 (PMC10533896; doi:10.1038/s41598-023-43218-3)
Supplement: Supplementary file 1 — Supplementary Figures. [file 41598_2023_43218_MOESM1_ESM.pdf]

Supplemental figure legend

Figure S1. Higher expression of CSE1L correlates with poorer outcomes in NSCLC patients. Overall survival (OS) in 2166 cases of NSCLC patients was analyzed using the KM-plotter database (https://kmplot.com). Two different probes of CSE1L (210766_s_at and 201112_s_at) showed higher levels of CSE1L significantly correlated with decreased OS in the patients.

Figure S2. There is no HDAC1 subcellular localization difference between control siRNA and CSE1L siRNA treated A549 cells. A549 p53+/+ and A549 p53-/- cells were transfected with control siRNA or CSE1L siRNA for 2 days. P53+/+ cells were separated into cytoplasmic (CY) and nuclear (NU) fractions and immunoblotted for the indicated proteins (A). Histone 3 (H3) is used as a marker for nuclear fraction. Total cell lysates of p53+/+ and p53-/- cells were immunoblotted for pRB1 and RB1 proteins (B). Original blots were presented on the right side.

Figure S3. Original blots for figure 2C. Images from three independent experiments are presented. Images from experiment 1 was used in figure 2C.

Figure S4. Original blots for figure 4A. Images from three independent experiments are presented. Images from experiment 1 was used in figure 4A.

Figure S5. Original histograms of FACS analysis of cell cycle for figure 3B and 3D.

Figure S6. Original histograms of FACS analysis of cell cycle for figure 4C.

Figure S7. Synergistic effect of mocetinostat and paclitaxel in A549 p53+/+ cells. The drug combination effect in figure 5D is analyzed with CompuSyn software and the results for A549 p53+/+ cells (A) and A549 p53-/- cells (B) were presented. Note that the combination index (CI) values <1 indicates synergy.

Figure S1

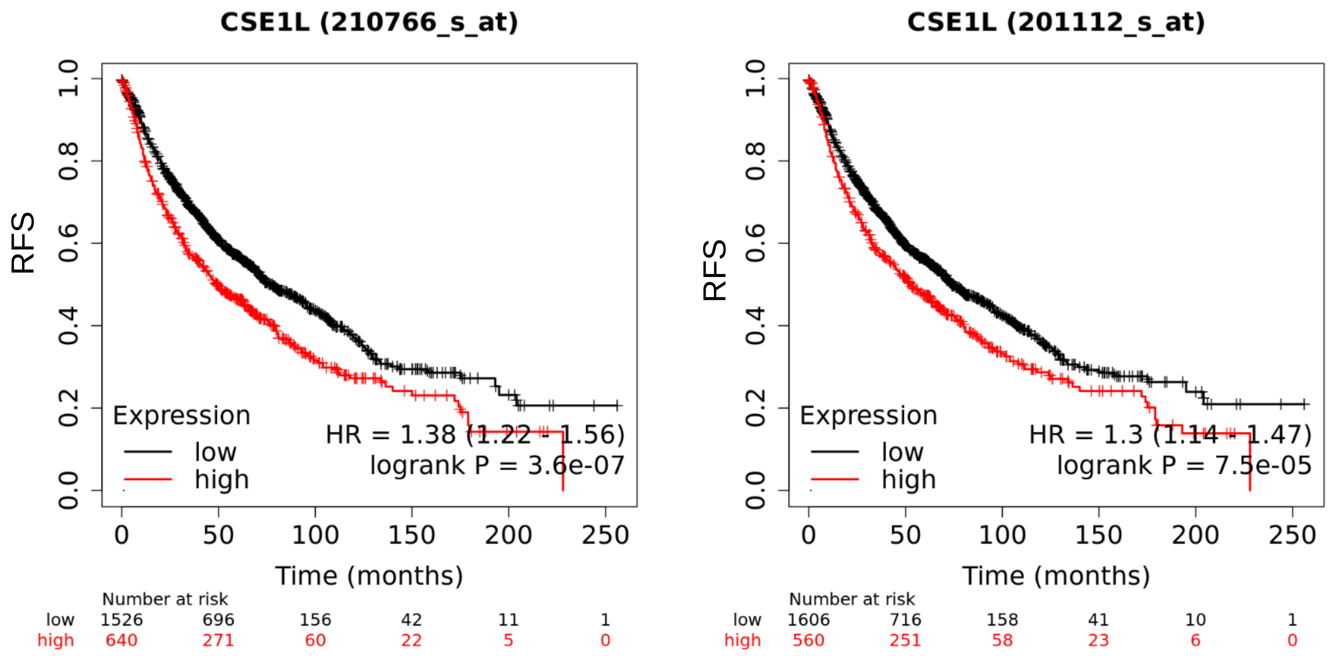

CSE1L (210766_s_at)
RFS
1.0
0.8
0.6
0.4
0.2
0.0
Expression
low
high
HR = 1.38 (1.22 − 1.56)
logrank P = 3.6e-07
0
50
100
150
200
250
Time (months)
Number at risk
low 1526 696 156 42 11 1
high 640 271 60 22 5 0
CSE1L (201112_s_at)
RFS
Expression
low
high
HR = 1.3 (1.14 − 1.47)
logrank P = 7.5e-05
Time (months)
Number at risk
low 1606 716 158 41 10 1
high 560 251 58 23 6 0

Figure S2.

A.

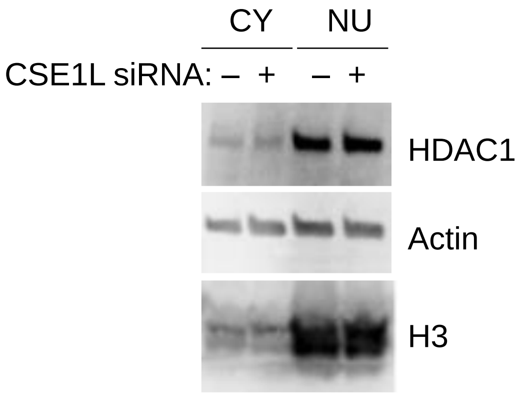

B.

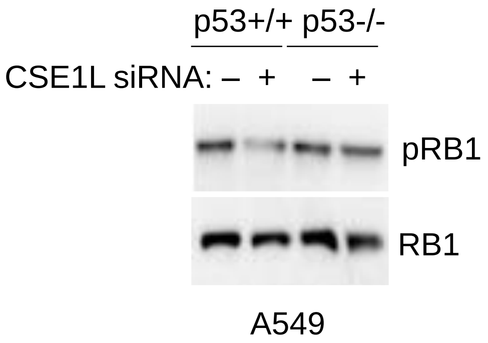

Original blots.

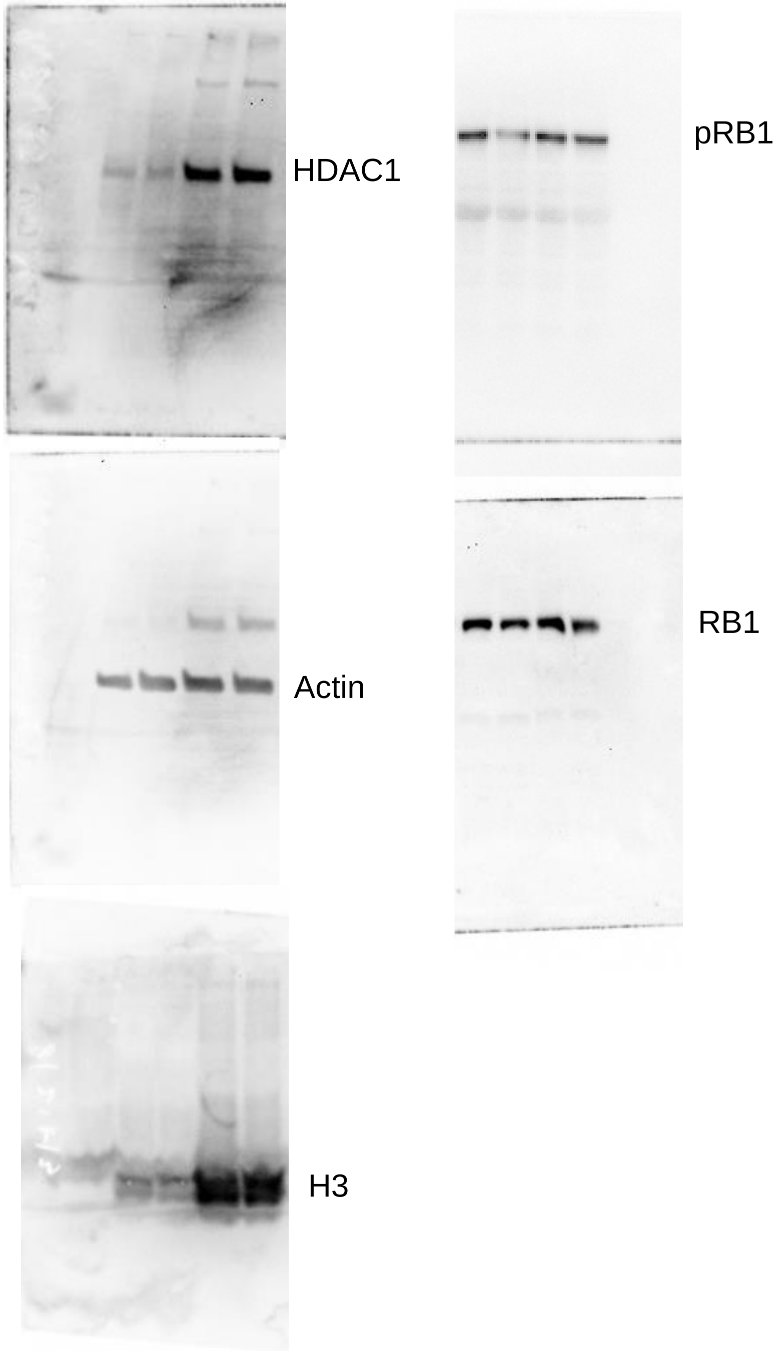

Figure S3

Original blots for figure 2C

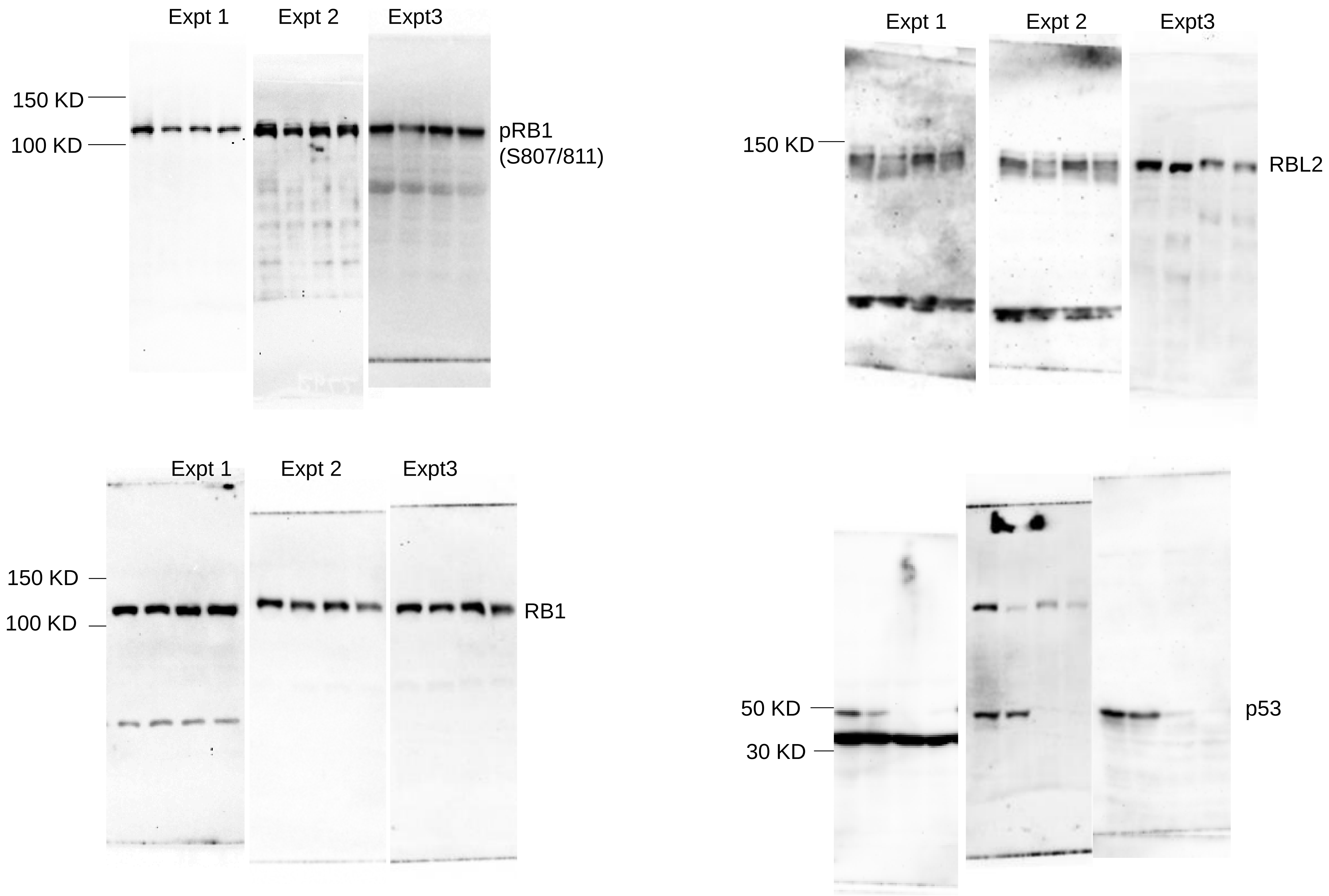

Figure S3-continue

Original blots for figure 2C

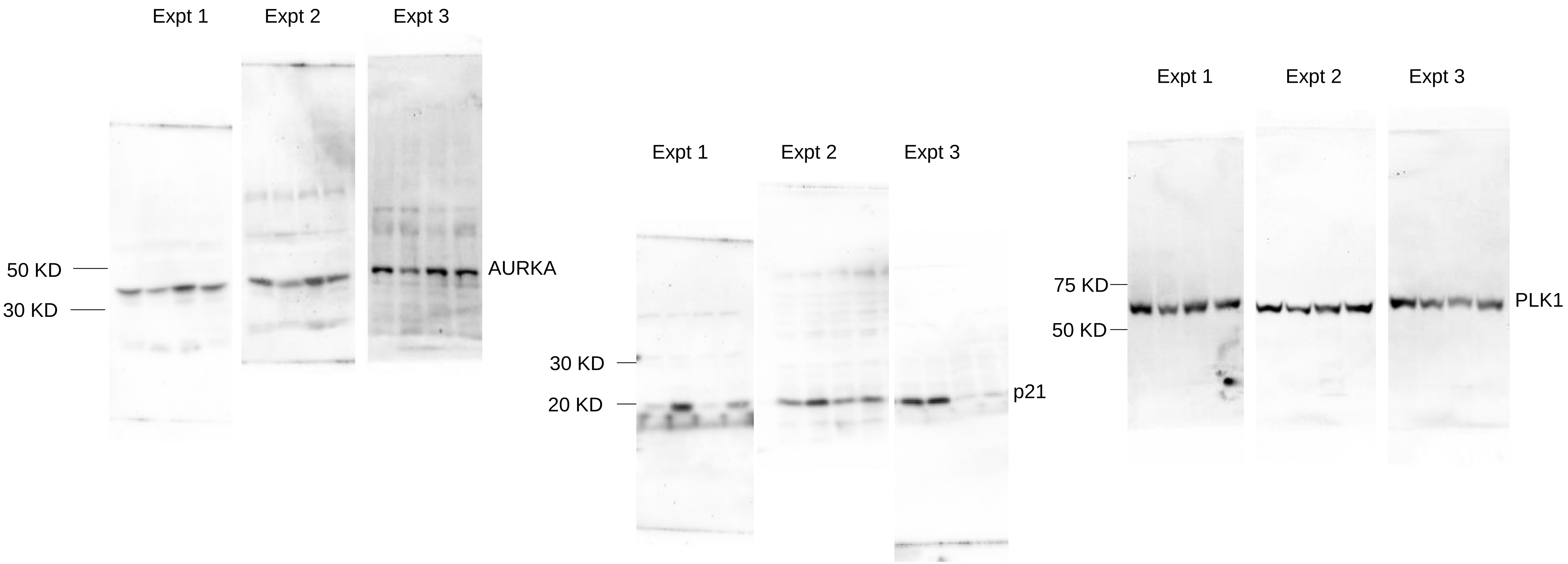

Figure S4

Original blots for figure 4A

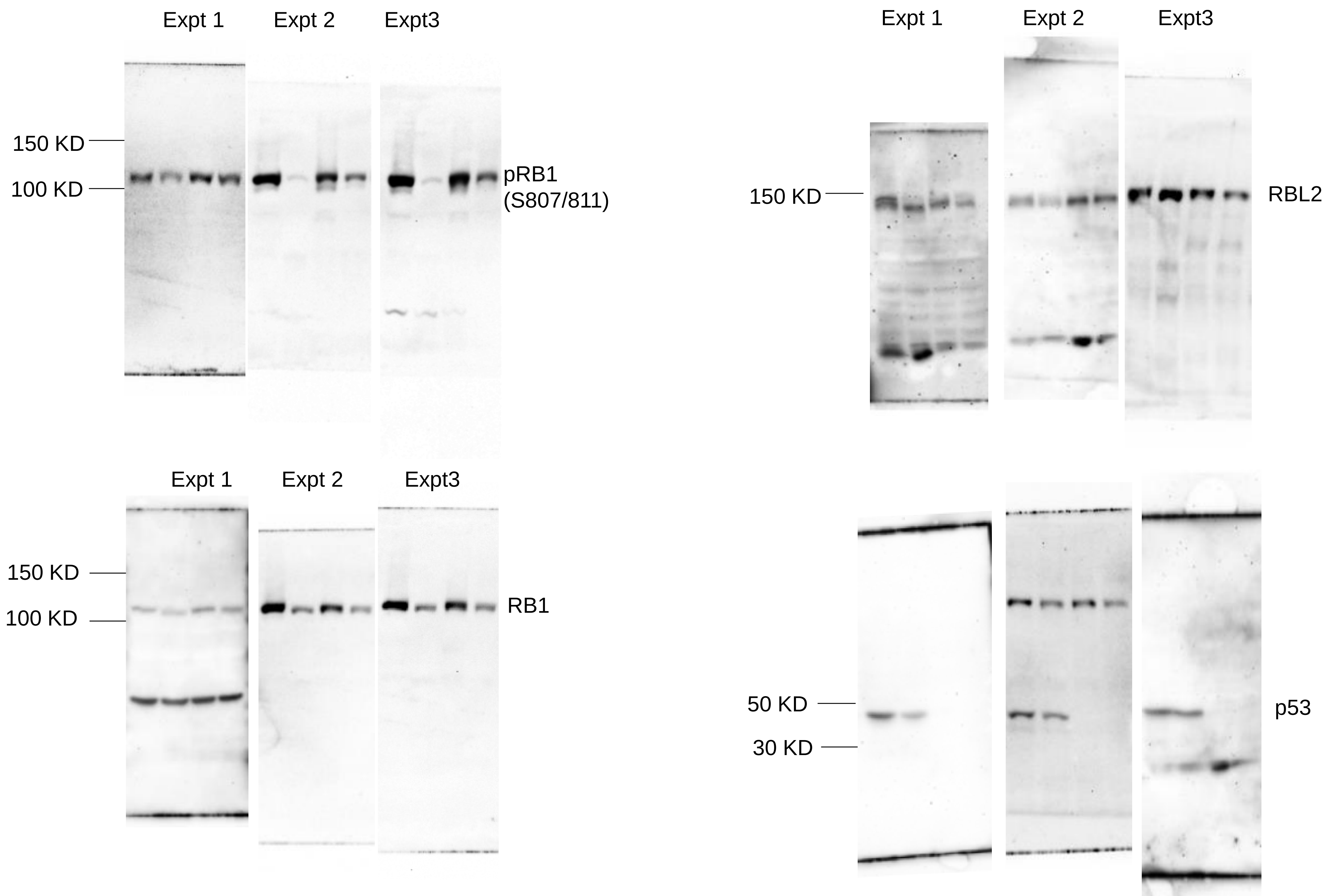

Figure S4-continue

Original blots for figure 4A

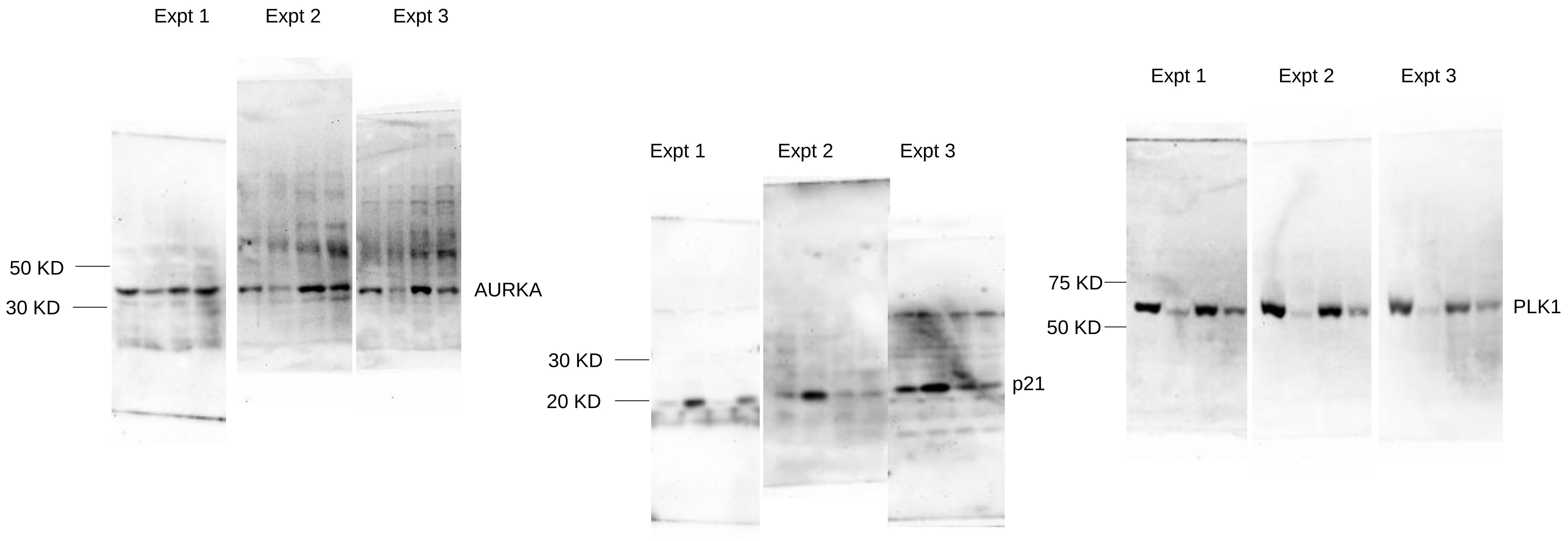

Figure S5: Original FACS analysis of cell cycle for figure 3B and 3D

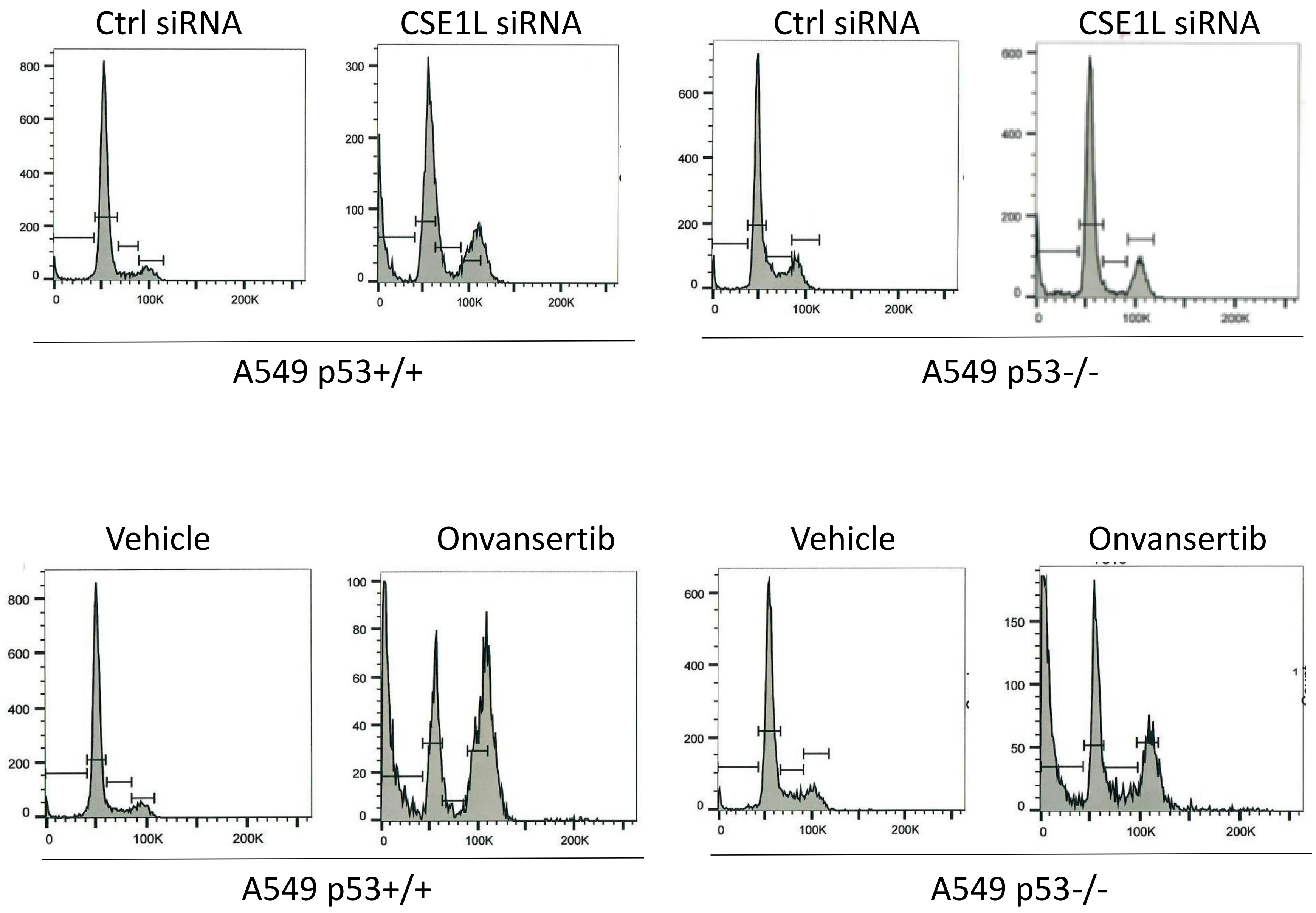

Figure S6: Original FACS analysis of cell cycle for figure 4C

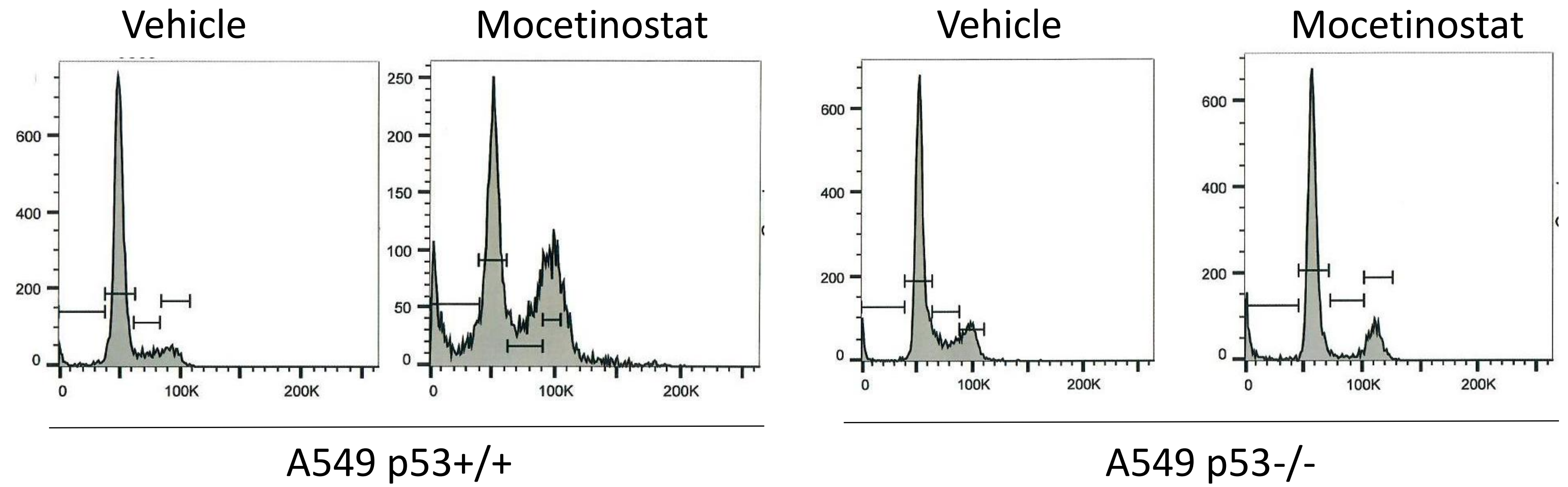

Figure S7

A,.

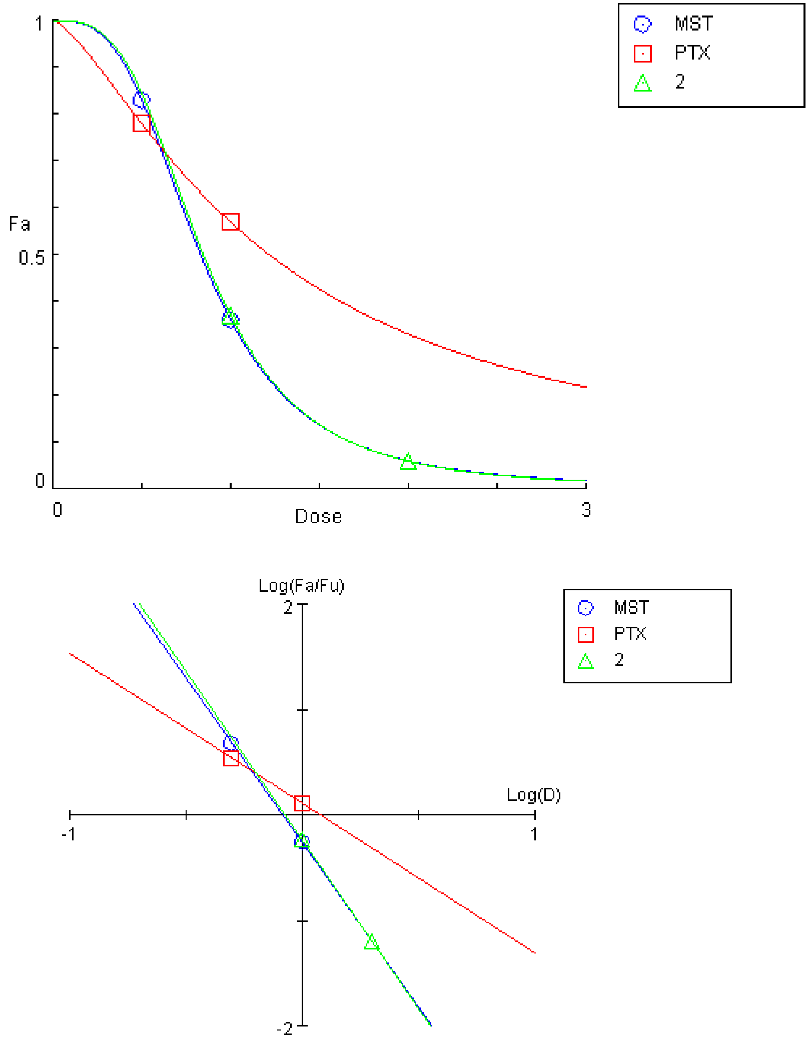

B.

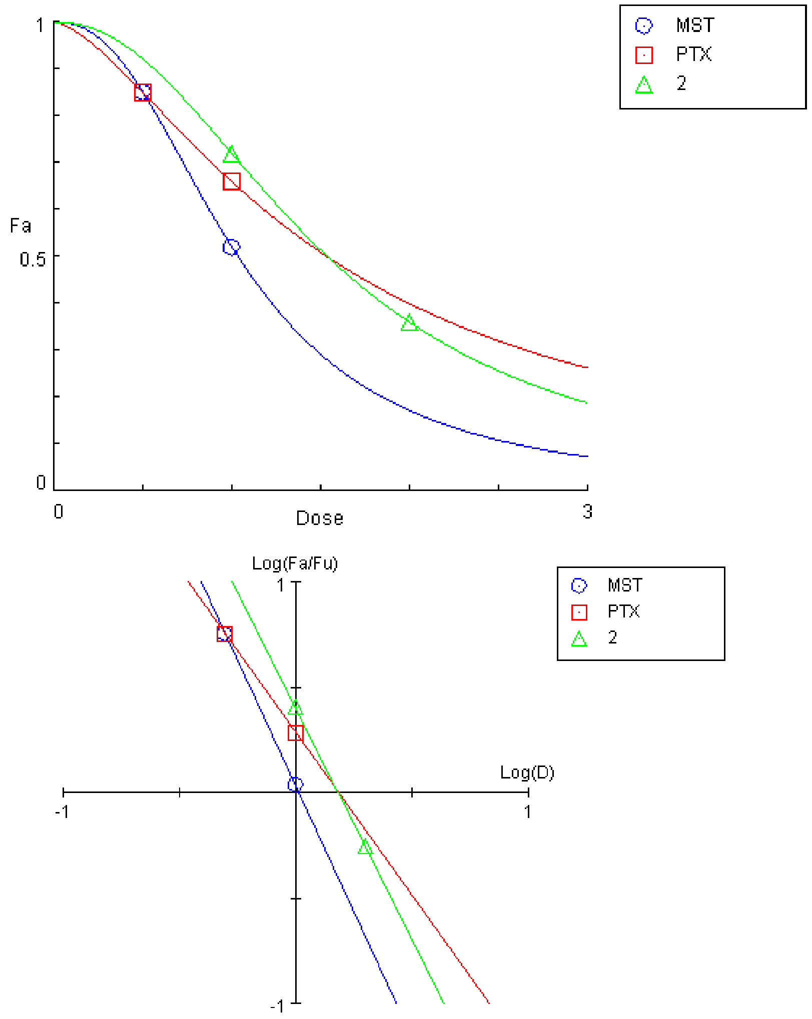

CI values for MST+PTX in A549 p53+/+ cells:

Total Dose	Fa	CI Value
MST 0.5+PTX 0.5	0.37	0.78873
MST 1+PTX1	0.06	0.61556

CI values for MST+PTX in A549 p53-/- cells:

Total Dose	Fa	CI Value
MST 0.5+PTX 0.5	0.72	1.31795
MST 1+PTX1	0.36	1.20859
